# Paradigm Shift to Neuroimmunomodulation for Translational Neuroprotection in Stroke

**DOI:** 10.3389/fnins.2018.00241

**Published:** 2018-04-10

**Authors:** Diana Amantea, Rosaria Greco, Giuseppe Micieli, Giacinto Bagetta

**Affiliations:** ^1^Section of Preclinical and Translational Pharmacology, Department of Pharmacy, Health and Nutritional Sciences, University of Calabria, Cosenza, Italy; ^2^Laboratory of Neurophysiology of Integrative Autonomic Systems, Headache Science Centre, IRCCS Mondino Foundation, Pavia, Italy; ^3^Department of Emergency Neurology, IRCCS Mondino Foundation, Pavia, Italy

**Keywords:** brain ischemia, immune system, inflammation, ischemic cascade, neuroprotection, stroke

## Abstract

The treatment of acute ischemic stroke is still an unresolved clinical problem since the only approved therapeutic intervention relies on early blood flow restoration through pharmacological thrombolysis, mechanical thrombus removal, or a combination of both strategies. Due to their numerous complications and to the narrow time-window for the intervention, only a minority of stroke patients can actually benefit from revascularization procedures, highlighting the urgent need of identifying novel strategies to prevent the progression of an irreversible damage in the ischemic penumbra. During the past three decades, the attempts to target the pathways implicated in the ischemic cascade (e.g., excitotoxicity, calcium channels overactivation, reactive oxygen species (ROS) production) have failed in the clinical setting. Based on a better understanding of the pathobiological mechanisms and on a critical reappraisal of most failed trials, numerous findings from animal studies have demonstrated that targeting the immune system may represent a promising approach to achieve neuroprotection in stroke. In particular, given the dualistic role of distinct components of both the innate and adaptive arms of the immune system, a strategic intervention should be aimed at establishing the right equilibrium between inflammatory and reparative mechanisms, taking into consideration their spatio-temporal recruitment after the ischemic insult. Thus, the application of immunomodulatory drugs and their ability to ameliorate outcomes deserve validation in patients with acute ischemic stroke.

## Introduction

Over the past three decades, experimental studies, as well as evidence from the clinical setting, have provided the basis for understanding the pathobiological mechanisms underlying stroke. Despite significant advances were made in the elucidation of cellular and molecular pathways implicated in brain ischemia, none of the previously identified potential molecular targets has actually been translated into an effective pharmacological therapy. Therefore, the concept of neuroprotection in stroke is increasingly considered as a chimera, leading to a strong disappointment in all the players involved in the research and development pipeline, from the financial supporters to the basic and clinical researchers. In fact, the only therapeutic intervention approved for the acute treatment of ischemic stroke patients is based on early blood flow restoration through pharmacological thrombolysis (within 4.5 h from symptoms onset), mechanical thrombus removal (within 6 h from onset) or a combination of both strategies (Saver et al., 2015; Goyal et al., 2016; Rodrigues et al., 2016; Campbell et al., 2017; Shireman et al., 2017). Two very recent clinical trials (DEFUSE3 and DAWN) have revealed that endovascular thrombectomy, initiated up to 16–24 h after patients were last known well, results in significant amelioration of outcomes, especially in patients with small infarct core volumes (i.e., with slow early DWI growth rate) (Albers et al., 2018; Nogueira et al., 2018). Thus, the paradigm “time is brain” deserves a revision, based on a better understanding of the evolution of ischemic core lesions and on imaging-based selection criteria of patients that have been suggested to underlie the “late window paradox” (Sandercock and Ricci, 2017; Albers, 2018).

Although the narrow time-window for the intervention might no longer be considered a major drawback of revascularization approaches (at least in some patients), these procedures are still endowed with a number of disadvantages, such as a relative high percentage of failures and a number of complications that strongly limit their therapeutic use (Gill et al., 2014; Saver et al., 2015; Balami et al., 2017; Kim, 2017). As a result, only a minority of stroke patients are eligible for revascularization procedures and can actually benefit from them. A major issue that needs to be considered relates to the fact that these approaches do not act on the brain tissue to provide neuroprotection, as they do not interfere with the cascade of events that lead to cerebral damage, neither they affect any process that prompts neuronal survival. This highlights the need for a better understanding of the cellular and molecular mechanisms involved in the development of a structural lesion in the ischemic penumbra, where the progression of an irreversible lesion occurs more slowly as compared to the rapidly demising core (Dirnagl et al., 1999; Heiss, 2012; Davis and Donnan, 2014). Reducing the growth rate of the ischemic core represents a pivotal pharmacological goal both to limit the progression of ischemic brain damage and to increase patient eligibility and success rate of endovascular procedures (Lo and Ning, 2016; Albers, 2018).

## From neuroprotection to immunomodulation

Cerebral ischemia is typically triggered by the interruption of blood supply to the brain, leading to energy failure, and activation of a cascade of events that ultimately causes brain damage. The ischemic cascade has been thoroughly described elsewhere (Dirnagl et al., 1999; Candelario-Jalil, 2009; Moskowitz et al., 2010; Heiss, 2012). Briefly, as a result of decreased oxygen supply, neurons are unable to accomplish aerobic respiration in mitochondria, intracellular pH decreases, deterioration of membrane ion gradients occurs, and cellular swelling results in cytotoxic oedema (Kohno et al., 1995; Hu and Song, 2017; von Kummer and Dzialowski, 2017). Excitatory neurotransmitters are released in the extracellular space, reaching concentrations that are toxic to neurons. Excitotoxicity, Ca^2+^-dependent activation of detrimental enzymes, excessive production of reactive oxygen species (ROS) and inflammation represent crucial mechanisms underlying blood-brain barrier (BBB) disruption and neuronal death in the ischemic brain (Benveniste et al., 1984; Rothman and Olney, 1986; Butcher et al., 1990; Bano and Nicotera, 2007; Anrather and Iadecola, 2016; Curcio et al., 2016; Amantea and Bagetta, 2017). At least in experimental model systems, the ischemic insult results in upregulation of diverse programmed cell death pathways, whereby the active crosstalk between apoptosis, necroptosis, and autophagy pathways ultimately affects cellular fate (Yuan and Yankner, 2000; Wang et al., 2018).

During the past three decades, in order to achieve neuroprotection, a large number of studies was aimed at validating these mechanisms as potential therapeutic targets. Despite effective neuroprotection was obtained in the preclinical setting by a number of approaches (i.e., glutamate receptor antagonism, calcium channel blockade, magnesium infusion, free radical scavenging, attenuation of inflammatory responses), there was an overwhelming failure to validate in patients these apparently promising findings. Several reasons have been postulated to explain why neuroprotection has not worked in human stroke, including limitations of the animal models used, erroneous preclinical or clinical trial design and/or inadequate selection of patients (Klehmet et al., 1999; Ford, 2009; Howells et al., 2014). Moreover, most of the studies on neuroprotection in stroke focused on acute mechanisms, occurring quite early after stroke injury, and were primarily aimed at targeting neuron-specific responses (Ginsberg, 2008). During the past decade, a more integrated view of the brain has highlighted the pivotal role of several components of the neurovascular unit in neuronal function and dysfunction (Iadecola, 2017). In fact, a dynamic crosstalk between neurons, glia, endothelium, and blood-borne cells dramatically affects the progression of ischemic brain damage (Lo and Ning, 2016). Due to the discovery that inflammatory mediators play a crucial role in the progression of the damage in the penumbra, more recently, the efforts of stroke researchers were devoted to the identification of neuroprotective candidates through the attenuation of the neuroinflammatory response (Veltkamp and Gill, 2016). In this context, hypothermia has been reported to decrease activation/production of inflammatory mediators in the ischemic brain (Deng et al., 2003; Lee et al., 2016; Sandu et al., 2016). However, the pure blockade of a single inflammatory mechanism has led to disappointing results, being most mediators endowed with dualistic effects on the progression of ischemic brain damage (Amantea et al., 2014). This is consistent with the ability of the brain to trigger regenerative responses that are essential for spontaneous recovery and involve cell genesis, axon growth, and synaptic modulation (Chu et al., 2012; Hermann and Chopp, 2014; Felling and Song, 2015). In this context, astrocytes, microglia, and monocytes/macrophages are among the most potent modulators of brain repair/regeneration (Amantea et al., 2015; Liu and Chopp, 2016). Indeed, a dualistic nature has been ascribed to the immune system, since both the innate and adaptive responses triggered following an ischemic stroke consist of detrimental or beneficial/reparative components that differentially evolve depending on the spatiotemporal progression of tissue injury (Fumagalli et al., 2015; Gill and Veltkamp, 2016).

## The involvement of the immune system

**Figure 1:**
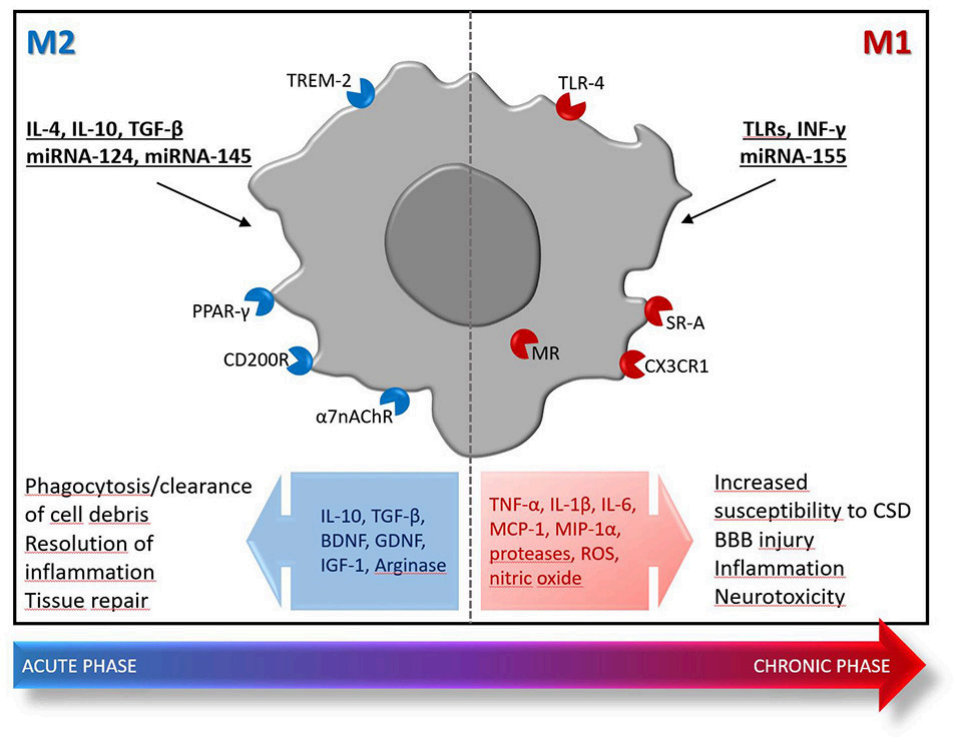
Polarization of microglia/macrophages toward protective (M2) or inflammatory (M1) phenotypes after ischemic stroke. Early after the ischemic insult, locally activated microglia/macrophages and blood-borne monocytes/macrophages display an M2 phenotype characterized by enhanced phagocytic activity and production of mediators [e.g., IL-10, transforming growth factor (TGF)-β, arginase] that provide resolution of inflammation and pro-survival effects toward hypoxic/ischemic neurons. This M2-mediated response is transient and quenches few days after the insult, being replaced by an inflammatory detrimental phase dominated by M1-polarized cells that exhibit reduced phagocytosis ability and release of inflammatory and neurotoxic mediators (e.g., TNF-α, IL-1β, IL-6, MCP-1, MIP-1α, proteases, ROS, nitric oxide). At later stages, release of regulatory and growth factors [e.g., brain-derived neurotrophic factor (BDNF), glia-derived neurotrophic factor (GDNF), and insulin-like growth factor (IGF)-1] by M2-like microglia/macrophages contributes to reparative mechanisms implicated in late tissue recovery. The M1/M2 dichotomy is an illustrative theoretical framework that only embodies two extreme activation states of a spectrum of different functional phenotypes that actually occur in the damaged tissue. The inflammatory M1 phenotypes are typically triggered by interferon (INF)-γ, TLRs modulation or miRNA-155; whereas, the reparative M2 phenotypes are prompted by IL-4, IL-10, TGF-β, miRNA-124, or miRNA-145. Moreover, a number of receptors have been demonstrated to trigger polarization toward the M2 or M1 phenotype, thus representing promising targets for successful immunomodulation in stroke: the α7 nicotinic acetylcholine receptor (α7 nAChR), the CD200R, the peroxisome proliferator-activated receptor-γ (PPARγ), the triggering receptor expressed on myeloid cells (TREM), the class A scavenger receptor (SR-A), the fractalkine receptor CX3CR1 and the mineralcorticoid receptor (MR).

Release of damage-associated molecular pattern molecules upon the ischemic insult, triggers a rapid activation and proliferation of local microglia (Li et al., 2013; Benakis et al., 2014) that acquires an amoeboid phenotype typically associated with phagocytosis that underlies the important task of clearing debris and repairing the tissue (Schilling et al., 2005; Fang et al., 2014; Li et al., 2015). However, microglia activation also results in the release of inflammatory mediators, such as tumor necrosis factor (TNF), and ROS that increase susceptibility to cortical spreading depression (CSD) (Shibata and Suzuki, 2017) and prompt BBB damage, thus fostering the brain recruitment of leukocytes, including monocytes, neutrophils, and T cells (Gelderblom et al., 2009; Chu et al., 2014; Ritzel et al., 2015). It has been suggested that stroke mobilizes immature Ly6C^hi^ inflammatory monocytes that infiltrate the ischemic brain early after injury, reaching the core of the lesion. Then, monocytes progressively acquire the expression of typical markers of alternatively activated M2 macrophages, like arginase-1 and Ym-1, suggesting their possible role in tissue repair during the sub-acute phase of stroke (Miró-Mur et al., 2015). At least in animal models, during the early phases after ischemia, microglia, and macrophages adopt an M2 reparative phenotype, then superseded by ischemia-induced M1-like phenotypes that populate the injured brain days after the initial insult (Figure 1; Perego et al., 2011; Hu et al., 2012; Fumagalli et al., 2015; Ritzel et al., 2015; Wattananit et al., 2016; Kronenberg et al., 2017; Greco et al., 2018). These inflammatory phenotypes participate to cerebral injury, by releasing neurotoxic substances [i.e., TNF-α, interleukin (IL)-1β, monocyte chemoattractant protein (MCP)-1, macrophage inflammatory protein (MIP)-1α, and IL-6] and ROS. To counteract this inflammatory, detrimental process, sublethally injured neurons in the ischemic penumbra produce IL-4, a potent M2-polarizing cytokine (Zhao et al., 2015). In a recent study, selective depletion of different monocyte/macrophage subsets did not influence functional and histological outcomes after 30 min of transient MCAo in rodents, thus suggesting that monocytes/macrophages may not affect damage after a mild ischemic insult (Schmidt et al., 2017). However, that study did not take into account that microglia and monocytes/macrophages experience mixed and complex polarization dynamics dramatically affected by the spatio-temporal production of ischemia-induced microenvironmental stimuli (Fumagalli et al., 2015). Understanding these mechanisms and identifying cellular targets that allow the fine tuning of M1-to-M2 polarization shift represent a promising strategy to implement clinical success of stroke therapy. This approach has been successfully validated in preclinical settings, whereby a number of drugs that reduce the M1/M2 ratio during the acute phase of stroke ameliorate histological and functional outcomes in rodents. Among these, there is evidence that certain antibacterial drugs endowed with strong immunomodulatory properties, such as minocycline and azithromycin, may represent promising candidates as stroke therapeutics (Liao et al., 2013; Yang et al., 2015; Amantea et al., 2016a,b). Similarly, other drugs belonging to different chemical and pharmacological classes share the ability to provide neuroprotection by reducing the M1/M2 ratio, including eplerenone spironolactone (Frieler et al., 2011, 2012), Exendin-4 (Darsalia et al., 2014), metformin (Jin et al., 2014), and rosiglitazone (Han et al., 2015). Intriguingly, the latter drug, as well as bexarotene, have been shown to provide neuroprotection in animal models of focal cerebral ischemia by promoting the polarization of neutrophils toward the beneficial N2 phenotype (Cuartero et al., 2013; Certo et al., 2015).

In fact, similar to other myeloid cells, neutrophils may display different features in stroke, ranging from the inflammatory functions of the N1 phenotype to supportive and beneficial roles for the N2 phenotype (Jickling et al., 2015; Ruhnau et al., 2017). After the ischemic insult, they rapidly release ROS and cytokines that, together with an increased activity of proteases, contribute to BBB rupture, brain oedema, and cerebral damage (Jickling et al., 2015; Frieler et al., 2017). Brain recruitment of neutrophils correlates with poor neurological outcome and brain damage severity both in humans and in rodents. Although there is some evidence arguing that they only accumulate in the perivascular space, without actually penetrating the brain parenchima (Enzmann et al., 2013; Perez-de-Puig et al., 2015), the crucial role of neutrophils in the progression of ischemic cerebral damage is clearly demonstrated (Matsuo et al., 1994; Garcia-Bonilla et al., 2014; Gelderblom et al., 2014; Maestrini et al., 2015; Neumann et al., 2015; Frieler et al., 2017). However, although neutrophils were regarded as promising pharmacological targets for the treatment of ischemic stroke, the success of clinical studies was limited by their beneficial properties (Jickling et al., 2015). Therefore, targeting myeloid cells by blocking their N1- or M1-mediated detrimental functions, while promoting their shift toward N2- or M2-like phenotypes at early times after injury should be considered a reliable strategy for neuroprotection in stroke.

## Evolution of innate and adaptive immune responses

A major advantage of targeting the immune system in ischemic stroke is ascribable to the possibility to widen the time-window for the intervention since immune responses are targetable in the acute, subacute, and chronic phases of recovery. The initial recruitment of local (i.e., microglia) and peripheral innate immune cells (i.e., monocytes/macrophages and neutrophils) underlies the early, non-specific, inflammatory response to the ischemic damage. Upon injury, the brain rapidly communicates with the periphery, thus an exponential increase in neutrophils count and an exponential decrease in the lymphocyte count occur in the hours immediately after stroke onset in patients (Veltkamp and Gill, 2016). Moreover, the abundance of inflammatory monocytes in the blood of stroke patients has been linked to unfavorable outcome (Urra et al., 2009; Kaito et al., 2013).

Genomic profiling of peripheral blood has allowed to identify critical genes and biological immune processes associated with ischemic brain injury in patients (Oh et al., 2012; Barr et al., 2015; Asano et al., 2016). The pathway of innate immunity toll-like receptors (TLRs) has been suggested to be upregulated in the blood of acute ischemic stroke patients within 24 h of symptoms onset (Barr et al., 2010), which is consistent with the evidence that poor outcome is associated with increased expression of TLR-4 in monocytes (Yang et al., 2008; Urra et al., 2009). Accordingly, TLR-4 contributes to brain damage and inflammation in mice subjected to focal cerebral ischemia and promotes haemorrhagic transformation induced by delayed tPA administration (Caso et al., 2007; García-Culebras et al., 2017). At later time-points, i.e., 24–48 h after stroke, the primary pathway expressed in the peripheral blood of stroke patients relies on cytotoxic T-lymphocyte antigen 4 (CTLA4) (Barr et al., 2015), a costimulatory molecule expressed by activated T cells that serves as a negative modulator of adaptive immune cell functions (Buchbinder and Hodi, 2015). Therefore, T cells responsiveness decreases during the first 24–48 h after human stroke, which is consistent with the shift into a suppression state of the adaptive immune response (Miró-Mur et al., 2016). Interestingly, innate immune responses have been shown to have a role in self-tolerance, since the soluble form of CD163, a scavenger receptor shed from the plasma membrane of activated monocytes after stroke, increases in the blood circulation upon injury and exerts inhibitory effects on lymphocytic activity and proliferation (Moeller et al., 2012; Buechler et al., 2013; Lee et al., 2014; O'Connell et al., 2017). Arginase 1, released from circulating neutrophils upon acute ischemic stroke, has also been suggested to contribute to lymphocyte suppression in patients (Asano et al., 2016; Petrone et al., 2016). Notably, stroke-induced immunodepression is characterized by a transient lymphopenia, lymphoid organ atrophy, and monocyte deactivation, a condition that serves to reduce the probability of an autoimmune response toward brain antigens exposed and presented to lymphoid cells after BBB disruption (Urra et al., 2014). However, any potential brain protective effect of stroke-induced immunodepression by attenuating or preventing lymphocyte-mediated brain damage is confounded by stroke severity and by an increased incidence of infections, that represents a major cause of death in the post-acute phase (Miró-Mur et al., 2016). Thus, the maintenance of an adequate equilibrium between self-tolerance and pathogen susceptibility is crucial for patient recovery, whereby the degree of immune suppression requires an adequate balance between innate and adaptive responses. In fact, the disruption of the crosstalk between innate and adaptive mechanisms is harmful and is predictive of poor recovery. As a consequence of this maladaptation, an elevated neutrophil/lymphocyte ratio is used to predict poor prognosis in acute ischemic stroke patients (Brooks et al., 2014; Celikbilek et al., 2014; Xue et al., 2017) and has recently been suggested to be an independent risk factor for ischemic stroke incidence in generally healthy adults (Suh et al., 2017).

The transient suppression of the immune response is followed by sensitization of the peripheral immune system to brain-derived antigens. These latter become unmasked by the insult, as rupture of the BBB and other mechanisms expose reactive peptides that are normally sequestered (Becker, 2009; Urra et al., 2014). By contrast, antigen-independent mechanisms are likely to underlie the deleterious effects of T cells in the very early phase of ischaemia (Gill and Veltkamp, 2016). Thus, an adaptive immune system response builds up through the recruitment of T lymphocytes and natural killer T cells within few days after stroke, followed by a delayed (i.e., persisting for up to 30 days after the insult) activation and proliferation of regulatory T cells (Treg) that restrain the inflammatory response triggered by acute brain damage (Gill and Veltkamp, 2016). Therefore, likewise the early innate immunity, late T-cell mediated responses are endowed with both inflammatory and protective functions. This may explain the clinical failure to reduce infarct growth of natalizumab, a humanized monoclonal antibody against the glycoprotein α4 integrin expressed on the surface of lymphocytes and monocytes (Elkins et al., 2017). By reducing the adhesion of leukocytes to the endothelial vessel wall, natalizumab blocks their brain infiltration in animal models, resulting in reduced infarct volume after the permanent distal occlusion of the middle cerebral artery, which causes a small cortical infarction (Llovera et al., 2015). The fact that, under certain experimental conditions, natalizumab failed to exert neuroprotection, despite its ability to reduce recruitment of T cells and neutrophils, can be explained on the basis of its specific mechanism of action that does not allow to discriminate different subpopulations of leukocytes, namely the drug also blocks cerebral invasion of potentially protective phenotypes (Langhauser et al., 2014; Tatlisumak, 2017). This further highlights that any intervention aimed at targeting the immune system should selectively suppress detrimental responses, while promoting those that contribute to resolution of inflammation, repair, and regeneration.

## Conclusions

Despite the numerous experimental efforts taken to identify potential pharmacological targets for neuroprotection in stroke, only little progress has been made in translating these findings to the clinical setting. As a consequence, patients can only rely on procedures that allow reperfusion of the occluded vessels, endowed with a number of limitations that maintain ischemic stroke, a leading cause of death and long-term disability worldwide. The results of two recent clinical trials (DEFUSE3 and DAWN) have deranged the “time is brain” paradigm, enhancing the expectations for eligibility of patients for endovascular thrombectomy, especially for those with a slowly expanding ischemic core. This will give new impulse to basic research, since blocking the mechanisms of infarct growth in the penumbra would result in better access to endovascular therapies, as well as in reduced brain damage and neurological deficit. Given the strict interplay between the ischemic penumbra and the innate and adaptive arms of the immune system, growing evidence highlights the promising neuroprotective effects of a rational immunomodulation in stroke models. Thus, re-establishing the equilibrium between inflammatory, detrimental immune responses, and reparative mechanisms represents a promising strategy that deserves validation in patients with acute ischemic stroke.

## Author contributions

All authors listed have made a substantial, direct, and intellectual contribution to the work, and approved it for publication.

### Conflict of interest statement

The authors declare that the research was conducted in the absence of any commercial or financial relationships that could be construed as a potential conflict of interest.
